# The effects of upper- vs. lower-body aerobic exercise on perceived pain in individuals with chronic knee pain: a randomised crossover trial

**DOI:** 10.3389/fpain.2023.1277482

**Published:** 2023-12-06

**Authors:** Rachel Deere, Enhad Chowdhury, Abby Tabor, Dylan Thompson, James L. J. Bilzon

**Affiliations:** ^1^ Centre for Clinical Rehabilitation and Exercise Medicine (CREM), Department for Health, University of Bath, Bath, United Kingdom; ^2^ Centre for Nutrition and Exercise Metabolism (CNEM), Department for Health, University of Bath, Bath, United Kingdom; ^3^ Centre for the Analysis of Motion, Entertainment Research and Applications (CAMERA), University of Bath, Bath, United Kingdom; ^4^ Centre for Trials Research, Cardiff University, Cardiff, United Kingdom; ^5^ Faculty of Health and Applied Sciences, University of West England, Bristol, United Kingdom

**Keywords:** chronic knee pain, musculoskeletal pain, exercise, symptomatic pain, experimental pain, exercise medicine, pain management

## Abstract

**Background and objectives:**

Some patients with chronic knee pain experience an increase in knee pain following a single bout of exercise involving their knee joint, which can negatively affect exercise adherence and thus result in reduced overall health and lack of disease management. We want to determine whether a single bout of upper-body (UB) aerobic arm-ergometry exercise is effective in reducing the experience of pain in those with chronic knee pain compared with lower-body (LB) aerobic leg ergometry exercise.

**Methods:**

A total of 19 individuals (women = 11, men = 8; age = 63 ± 8 years; body mass index = 24 ± 3 kg/m^2^) who suffered from chronic knee pain for ≥3 months took part in this study. Arm-ergometry and cycle-ergometry exercises were performed for 30 min at a moderate intensity, separated by 7 days. Pain intensity was assessed by means of a visual analogue scale (VAS) pre- and post-exercise and for 7 days post-exercise. Pressure pain threshold (PPT) and mechanical detection threshold (MDT) were measured pre- and post-exercise at both local and distal anatomical sites. Data are presented as mean ± SD.

**Results:**

VAS pain was significantly reduced (*p* = 0.035) at 1 day post-exercise following the UB exercise trial (−1.4 ± 0.8) when compared with the LB exercise trial (+0.1 ± 2.1). Both UB and LB exercises were effective in reducing local and distal PPT. MDT responses were heterogeneous, and no differences between the UB and LB exercise conditions were noted.

**Conclusion:**

An acute bout of upper-body aerobic arm-ergometry exercise evoked a significant decrease in the affected knee joint pain in individuals with chronic knee pain of up to 24 h/1 day post-exercise compared with lower-body aerobic exercise. While the exact mechanisms remain unclear, upper-body exercise may offer a viable, novel therapeutic treatment for patients with chronic knee pain.

## 1. Introduction

Exercise is recommended as one of the primary therapeutic treatments for the conservative management of knee osteoarthritis (OA) (1). However, it is common for individuals to experience an acute increase in pain following a single bout of lower-body exercise, occurring from immediately post-exercise up to 1 day post-exercise (2–4). These undesirable symptoms have potential implications for adherence and compliance to exercise training, acting as a barrier for those with knee OA to meet minimum physical activity guidelines (5).

Despite this, evidence suggests that exercise training reduces the size of acute exercise pain responses (2), as well as overall knee pain (6, 7). As such, determining the efficacy of alternative exercise strategies that will reduce or eradicate acute increases in pain following a single bout of exercise, while being beneficial for chronic knee pain, is warranted. Exercise not involving the affected knee joint may serve as a short-term (or long-term) alternative to joint-specific exercise for knee pain relief. This may particularly be useful when severe pain is being reported in at least one knee joint, and exercise involving the affected joint is not possible or desirable from a patient and clinical perspective. Determining an alternative exercise modality for pain relief for individuals with chronic knee pain may also improve adherence to exercise, helping with disease management and improving overall health.

Two previous studies have compared the pain-relieving effects of exercise involving and not involving the affected knee joint. One compared a single bout of upper- (UB) and lower-body (LB) resistance exercise at 60% of 1 repetition max (1RM) in 11 individuals with knee OA (8), and although a positive effect of UB exercise on pain was observed (Cohen's *d* = 0.84), this pain was experimentally induced, and no measures of symptomatic pain which is a clinically fundamental outcome measure in this population were reported (9). The other study (10) looked at the effects of 4 weeks of LB resistance exercise training combined with moderate-intensity aerobic arm-ergometry or treadmill exercise in 78 patients with knee OA. Positive effects were reported for pain and function in the arm-ergometry group, in comparison with treadmill exercise (partial eta squared = 0.116). However, it is not possible to discern whether these improvements were related to the combination of LB resistance exercise and arm ergometry or a single component of the exercise intervention.

Our objective was to determine whether a single bout of UB aerobic exercise was effective in reducing the experience of pain in patients with chronic knee pain compared with LB aerobic exercise. Our hypothesis was that a greater, positive change is expected in the pain experience from baseline to 1 day post-exercise, following UB aerobic exercise compared with LB exercise. We based this hypothesis on the outcomes of the previous research discussed above and evidence surrounding the superiority of aerobic exercise on pain in this population compared with other modalities (11), coupled with the avoidance of using the affected joint and therefore reducing the possibility of an acute increase in knee pain.

## 2. Methods

### 2.1. Study population

Men and women aged between 45 and 69 years were recruited for the study. Participants were eligible to take part if they have suffered from chronic knee pain for >3 months (either uni- and bi-laterally), have no joint-related morning stiffness or morning stiffness lasting less than 30 min, and suffer from activity-related joint pain. Those who reported to have previously diagnosed with clinical knee OA were also included, as long as they also met the other inclusion criteria. The exclusion criteria included joint-specific injury within the last 6 months and OA or another chronic pain condition at any upper-body sites.

### 2.2. Study design and protocol

This was a randomised crossover experimental study. The trial was registered with ClinicalTrials.Gov (NCT05315934) and received a favourable ethics opinion from the Research Ethics Approval Committee for Health (REACH) at the University of Bath (EP 18/19 088). A random allocation sequence was performed by an individual outside of the research team after the participants’ first visit, using a random-number table. Informed written consent was obtained from all participants before the test. The participants attended the research laboratory on four separate occasions. During the first visit, the participants completed an arm-ergometry (12) and cycle-ergometry (13) perceptually graded exercise test (PGET) in order to predict $\dot{V}O_{2}$ peak (14) and prescribe the exercise intensity for the exercise trials. Each PGET consisted of four stages lasting for 5 min each, maintaining incremental intensities of 9, 11, 13, and 15 rating of perceived exertion (RPE) throughout each stage. Expired air was collected via the Douglas bag method, and heart rate (HR) was measured for the final minute of each stage. At least 7 days were left before the second visit. During the second visit, the participants completed a 20-min familiarisation of both UB and LB exercises so that relative exercise intensity could be adjusted for the main trial days if required. HR was tracked during these familiarisation sessions, but no measures were taken. At least 7 days were left before the third visit. During the third and fourth visits, participants completed the UB and LB exercise in a randomised order, separated by exactly 7 days. The arm-ergometry and cycle-ergometry exercise bouts were performed for 30 min at a moderate intensity corresponding to RPE13 [46%–63% $\dot{V}O_{2}$ peak (15)]. Expired air was collected using the Douglas bag method, and HR was measured at minutes 9–10, 19–20, and 29–30.

### 2.3. Outcome measures

#### 2.3.1. Primary outcome

To measure the symptomatic pain intensity of the participants immediately pre- and immediately post-exercise and at 24 h to 7 days following each trial visit, we employed a 0–10 visual analogue scale (VAS) (where 0 indicates no pain and 10 indicates the worst pain possible). The participants were required to rate their current pain intensity on VAS in their affected knee(s). The VAS was administered at the same time of the day across both the UB and LB trial days, and the participants rated their pain by pointing to the number that represented their pain level at that time. The VAS has recently been determined to be the most reliable measure of knee OA pain intensity compared with numerical rating scales and verbal rating scales (16).

#### 2.3.2. Secondary outcomes

Two quantitative sensory testing (QST) measures were employed as indicators of mechanical hyperalgesia and peripheral and central pain sensitisation. Mechanical detection threshold (MDT) and pressure pain threshold (PPT) were measured pre- and post-exercise at six anatomical locations: the extensor carpi radialis longus, the rectus femoris, and the medial joint line of the knee, on both the index and other side of the body (index = the side of the body where the affected knee is; other = the side of the body where there is no or less knee pain). The inclusion of MDT as a measure of mechanical hyperalgesia was exploratory, as limited data in this population currently exist. PPT has been more commonly utilised and measured in this population as an indicator of sensitisation (17), and more evidence is available supporting the fact that individuals with chronic knee pain and knee OA exhibit lower PPTs than healthy counterparts (17, 18):

I. MDT measures responses to innocuous stimuli. MDT was measured using a standardised range of von Frey filaments (Aesthesio Precise Tactile Sensory Evaluator 20-piece Kit) which exert forces between 0.25 and 512 mN. Participants were asked to verbally signal when touch was felt. The “methods of limits” were used to determine five thresholds. The reported threshold was the geometric mean of the five series (19). The test–retest reliability for MDT in the knee OA population has demonstrated moderate variability (20).
II. PPT measures responses to noxious stimuli. PPT was measured using a handheld pressure algometer (FDX25, Wagner Instruments, USA) applied to the skin with a 1-cm diameter probe that exerts forces up to 500 N. Participants were asked to verbally signal the point at which the minimum pressure induces pain. The PPT was determined with three series of ascending stimulus intensities applied as a slowly increasing ramp (19) of approximately 5 kPa/s with 10 s of rest between series. The test–retest reliability for PPT in the knee OA population has demonstrated the least variability of all QST measures (20).

Psychological characteristics were evaluated using self-report questionnaires. The brief fear of movement scale (BFOM) for OA was used to assess fear of movement (21) at baseline. The scale consists of six questions, answered on a four-point scale from “strongly agree” to “strongly disagree”. Scores can range from 6 to 24 with higher scores indicating a higher fear of movement.

Mental health symptoms were measured using the arthritis impact measurement scale (AIMS) for anxiety and depression (22) at baseline. The scale has 10 questions divided into two subscales (tension *n* = 5 and mood *n* = 5) both answered on a five-point scale from “always” to “never.” Scores can range from 10 to 25 for each subscale and are normalised to determine a score out of 10 for each subscale. Higher scores indicate greater levels of anxiety and depression.

The knee injury and osteoarthritis outcome score (KOOS) was used to assess the opinion of the participants about their knee and associated problems including subscales for pain, other symptoms, function in daily living (ADL), function in sport and recreation, and knee-related quality of life (QoL) (23). A Likert scale ranging from 0 (no problems) to 4 (extreme problems) is used for all subscales with scores calculated as the sum of the items included. Scores are transformed to a 0–100 scale with lower scores indicating more severe problems. The KOOS scale was measured at pre-exercise on both trials.

### 2.4. Statistical analysis and sample size

All in-text values are reported as mean ± SD, with significance-level set at *p* < 0.05, unless stated otherwise. All statistical tests were performed using IBM SPSS. All data were normally distributed and determined by Shapiro–Wilk analysis. Two-way (condition × time) repeated measures analysis of variance (ANOVA) tests were used to compare VAS scores from pre-exercise to post-exercise and 24 h/1 day post-exercise. *Post-hoc* Bonferroni corrections were applied to allow for multiple comparisons. An additional separate two-way ANOVA test was used to assess changes in VAS pain from 1 to 7 days post-exercise, for exploratory purposes. Two-way ANOVA tests were also performed to compare changes in MDT and PPT, at each anatomical site from pre- to post-exercise, with *post-hoc* Bonferroni corrections applied. Pearson's correlations were performed to explore relationships between baseline pain and the pain response to exercise. Pearson's correlations were also performed for exploratory purposes to identify potential relationships between BFOM, AIMS, and KOOS and the pain response (VAS, MDT, and PPT) to exercise. Sex differences in pain outcomes (VAS, MDT, and PPT) were assessed using a one-way ANOVA test. Trial order effects were also assessed for VAS, MDT, and PPT using a two-way ANOVA test (24, 25). Effect sizes (Cohen's *d*) were calculated for change in VAS pain from baseline (small effect = 0.20–0.40; medium effect = 0.50–0.79; and large effect >0.80).

An *a priori* power calculation based upon a previous randomised crossover trial (8) for Cohen's *d* = 0.7, α = 0.05, β = 0.80 determined that we would require a total of 19 participants.

## 3. Results

### 3.1. Participant characteristics and exercise data

Participant characteristics and baseline measures are reported in Table 1. Twenty-one individuals were screened, and complete data were available and included for 19 participants. Two participants dropped out due to COVID-19-related complications (after visit 2) and personal matters (after visit 1). A total of eight participants reported a clinical knee OA diagnosis, and 10 participants reported with bi-lateral chronic knee pain.

**Table 1:** Participant characteristics and baseline measures.

Allocation sequence	All	LB–UB	UB–LB
Sex (M/F)	8/11	4/4	4/7
Age (years)	63 ± 8	62 ± 9	64 ± 7
Body mass (kg)	82 ± 12	83.7 ± 13.9	80.9 ± 9.6
BMI (kg/m^2^)	24 ± 3	24.2 ± 4.0	23.3 ± 2.6
$\dot{V}O_{2}$ peak (ml/kg/min)	Arm ergometry—14.7 ± 4.3 Cycle ergometry—19.9 ± 5.6	15.9 ± 2.3 22.1 ± 5.3	13.8 ± 5.5 18.1 ± 5.7
KOOS symptoms (0–100)	59 ± 16	58 ± 13	59 ± 18
KOOS pain (0–100)	62 ± 14	57 ± 14	66 ± 13
KOOS ADL (0–100)	72 ± 14	72 ± 13	72 ± 14
KOOS sport/rec (0–100)	42 ± 17	44 ± 25	41 ± 12
KOOS QoL (0–100)	47 ± 18	44 ± 24	49 ± 14
BFOM (6–24)	12 ± 4	13 ± 4	12 ± 4
AIMS (0–10)	Tension—4.6 ± 1.1 Mood—7.4 ± 0.9	4.4 ± 1.1 7.4 ± 0.7	4.8 ± 1.1 7.5 ± 1.0

BMI, body mass index; $\dot{V}O_{2}$, oxygen uptake; KOOS, knee injury and osteoarthritis score; BFOM, brief fear of movement scale; AIMS, arthritis impact measurements scale.

**Figure 1:**
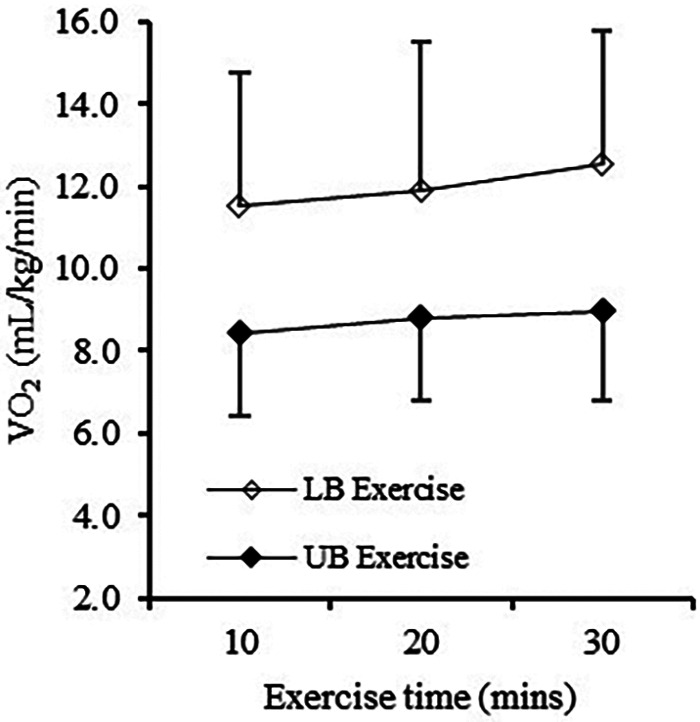
Relative $\dot{V}O_{2}$ values during both exercise trials.

The mean relative $\dot{V}O_{2}$ during the UB and LB trials were 8.7 ± 2.19 ml/kg/min (59.2% of $\dot{V}O_{2}$ peak) and 11.8 ± 3.4 ml/kg/min (59.3% of $\dot{V}O_{2}$ peak), respectively (Figure 1). HR and wattage averaged at 103 ± 11 bpm and 24 ± 8 W for the UB trial and 113 ± 16 bpm and 52 ± 21 W for the LB trial.

### 3.2. Primary outcome

**Figure 2:**
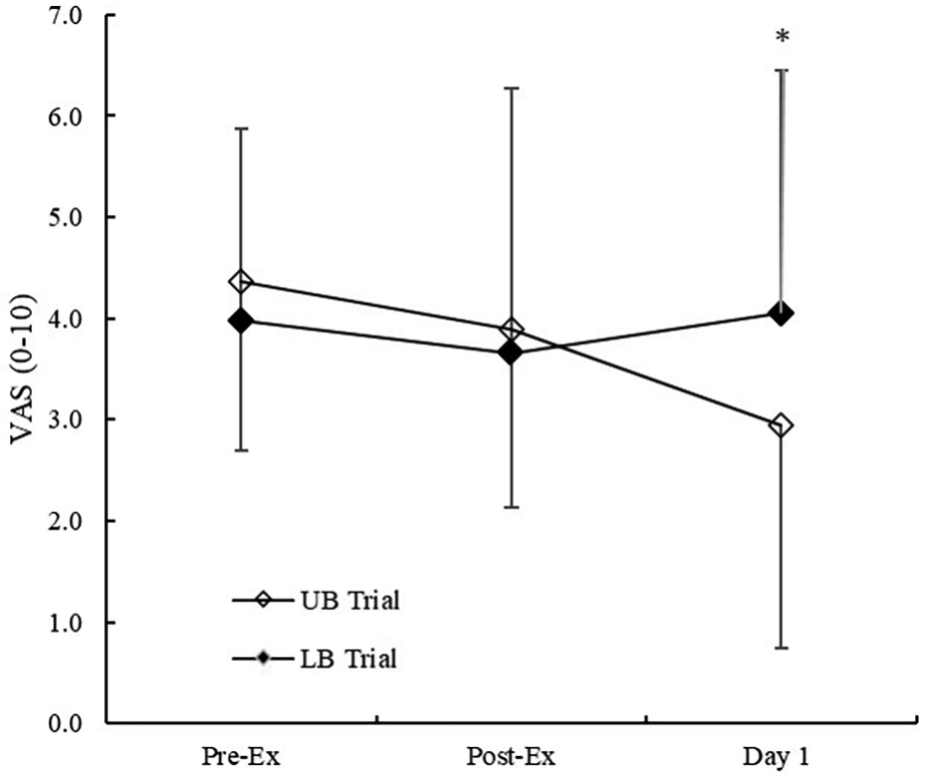
Absolute VAS scores from pre-exercise to 24 h/1 day post-exercise.

Where absolute values were analysed, a significant interaction effect between exercise conditions and VAS scores was observed (*p* = 0.035). VAS scores did not differ between trials immediately post-exercise (*p* = 0.723); however at 24 h/1 day post-exercise, VAS scores were significantly reduced following the UB trial (*p* = 0.002) but remained unchanged from baseline following the LB trial (*p* = 0.875) (Figure 2).

### 3.3. Secondary outcomes

#### 3.3.1. MDT

Mean MDT data are presented in Table 2. No significant time (*p* = 0.198), trial (*p* = 0.065), anatomical side (*p* = 0.364), or interaction effects (*p* = 0.723) were reported for MDT at the forearm. A significant effect of time (*p* = 0.005) was seen for MDT at the quadriceps. *Post-hoc* analysis determined that UB exercise improved MDT at the index quadriceps (*p* = 0.002) but not at the other quadriceps (*p* = 0.113), and LB exercise improved MDT at the other quadriceps (*p* = 0.006) but not at the index quadriceps (*p* = 0.061). A significant interaction effect for side × time (*p* = 0.043) was noted for MDT at the knee following UB exercise. UB exercise had a positive effect on MDT at the index knee (*p* = 0.041) but not at the other knee (*p* = 0.971).

**Table 2:** Mean ± SD MDT and PPT values from both exercise trial days.

	Forearm		Quadriceps		Knee	
	Pre	Post	Pre	Post	Pre	Post
UB trial						
MDT index (mN)	0.04 ± 0.07	0.04 ± 0.08	0.69 ± 0.37	0.38 ± 0.19^a^	0.84 ± 0.56	0.51 ± 0.57^b^
MDT other (mN)	0.04 ± 0.08	0.02 ± 0.02	0.51 ± .065	0.27 ± 0.32	0.45 ± 0.37	0.46 ± 0.81
PPT index (N)	45.9 ± 26.8	56.7 ± 25.6^a,c^	86.4 ± 38.3	101.4 ± 40.1^a^	78.1 ± 43.9	86.2 ± 40.5^a^
PPT other (N)	49.1 ± 23.1	59.0 ± 21.7^a,c^	107.5 ± 43.4	121.5 ± 45.7^a^	89.0 ± 40.6	101.4 ± 39.1^a^
LB trial						
MDT index (mN)	0.04 ± 0.06	0.03 ± 0.05	0.66 ± 0.28	0.51 ± 0.49^a^	1.08 ± 0.65	1.02 ± 1.70
MDT other (mN)	0.03 ± 0.05	0.01 ± 0.01	0.33 ± 0.27	0.23 ± 0.30^a^	0.49 ± 0.39	0.50 ± .091
PPT index (N)	40.7 ± 22.5	45.1 ± 19.5^a^	82.5 ± 37.8	91.9 ± 37.8^a^	73.8 ± 43.6	80.3 ± 45.0^a^
PPT other (N)	44.1 ± 23.3	50.6 ± 25.1^a^	104.7 ± 45.9	115.5 ± 47.6^a^	89.6 ± 42.9	100.2 ± 47.0^a^

^a^ Represents a significant effect of time (*p* < 0.05).

^b^ Represents a significant effect of side (*p* < 0.05).

^c^ Represents a significant effect of trial (*p* < 0.05).

#### 3.3.2. PPT

Mean PPT data are presented in Table 2. There was a significant interaction effect for trial × side (*p* = 0.040) for PPT at the forearm. *Post-hoc* analysis determined that UB exercise improved PPT at the forearm on both the index (*p* < 0.001) and other side (*p* < 0.001), but LB exercise only improved PPT at the forearm on the other side (*p* = 0.018) and not on the index side (*p *= 0.138). A significant effect of time (*p* = 0.016) for PPT at the quadriceps was seen. Regardless of trial, and side of the body tested, exercise was effective in improving PPT at the quadriceps (UB index *p* < 0.001; UB other *p* < 0.001; LB index *p* < 0.001; LB other *p* = 0.018). A significant effect of time (*p* = 0.016) for PPT at the knee was also observed. Regardless of trial and side of the body tested, exercise was effective in improving PPT at the knee (UB index *p* = 0.042; UB other *p* < 0.001; LB index *p* = 0.028; LB other *p* < 0.001).

### 3.4. Correlations

**Figure 3:**
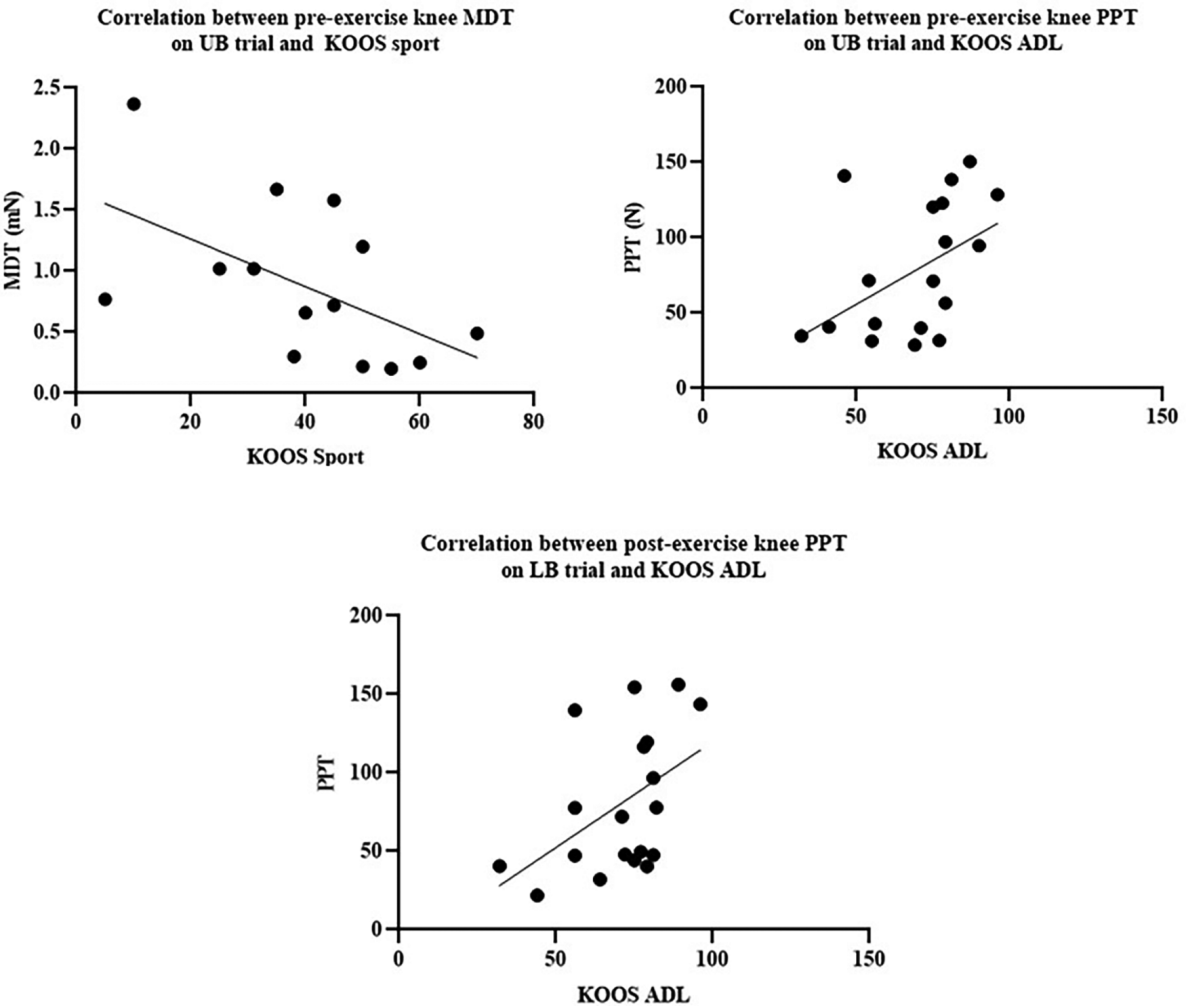
(**A**) Correlation between pre-exercise knee MDT on UB trial and KOOS sport. (**B**) Correlation between pre-exercise knee PPT on UB trial and KOOS ADL. (**C**) Correlation between post-exercise knee PPT on LB trial and KOOS ADL.

A moderate negative correlation between baseline MDT at the index knee and KOOS sport/rec score on the UB trial was identified (*r* = −0.544, Figure 3A).

A moderate positive correlation between baseline PPT at the index knee and KOOS ADL score on the UB trial (*r* = 0.457) was determined (Figure 3B). A moderate positive correlation between post-LB exercise PPT at the knee and KOOS ADL score (*r* = 0.474) was also identified (Figure 3C).

### 3.5. Sex differences and trial order effects

No sex differences were reported in VAS responses on neither the UB (*p* = 0.911) nor LB (*p* = 0.459) exercise trials. Similarly, no sex-related differences were found in MDT and PPT responses across both trials.

Analysis of order × condition interactions revealed no effect of the first trial on the observed effect of VAS pain (*p* = 0.423). This was reflected for MDT (*p*-value ranges from 0.458 to 0.948) and PPT outcomes (*p*-value ranges from 0.156 to 0.675).

## 4. Discussion

This study examined the effects of a single bout of either upper or lower-body aerobic exercise on the experience of pain in individuals with chronic knee pain. The primary outcome of the study was VAS pain. The results of this study revealed that a single acute bout of upper-body arm-ergometry exercise is more effective than a single acute bout of lower-body cycle-ergometry exercise in reducing symptomatic pain in the knee from immediately pre-exercise to 1 day post-exercise, measured on a VAS in our individuals with chronic knee pain. It is not clear whether these effects can persist for more than 24 h/1 day.

Lower-body exercise has previously been demonstrated to cause acute knee pain flares in some, but not all, patients with knee OA (2, 7). Although the exact mechanisms that evoke this acute pain response have not been discerned, it has been suggested that joint loading may be an influential factor (3, 26). This could, in part, explain why the UB exercise protocol used in our study evoked a positive pain response in comparison to the LB exercise, through avoiding load on the knee joint (26). In addition, physiological factors (e.g., systemic reduction in inflammation) (27, 28) may play a role. Aerobic exercise acutely increases systemic norepinephrine (29), which has previously shown to reduce both microglia proliferation and the release of pro-inflammatory cytokines from microglial cells throughout the brain and spinal cord and into systemic circulation (30). Microglial cell activation has been associated with a range of chronic pain conditions, but specifically, murine OA models have advocated that persistent pain is associated with microglial cell activation (31, 32). Aerobic exercise also acutely increases systemic IL-6 concentrations (33), enhancing IL-10 and IL-1ra (34), all of which possess anti-inflammatory properties. This combination of peripheral drivers and physiological factors may provide a sound explanation for the positive pain response perceived following UB exercise protocol compared with LB exercise protocol.

Secondary outcomes of the study were focused on experimentally induced measures of pain. PPT increased following both UB exercise and LB exercise which is suggestive of a normal exercise-induced hypoalgesia (EIH) response in line with the previous research in both healthy populations (35) and knee OA populations (36). MDT responses to UB and LB exercise were mixed, but notably, knee MDT was only improved following UB exercise. The mechanism behind this difference is unclear, and no previous research was found to compare these findings. Some changes in pain sensitisation were observed at anatomical locations distal from the region activated during exercise. While this may be related to contextual factors, where participants exaggerate their perceptions of pain due to the nature of the study, it seems more likely that the exercise resulted in a whole body change in pain perception.

### 4.1. Clinical implications

The findings of this study may have significant clinical implications. The reduction in knee pain following UB exercise suggests that exercise avoiding the affected knee joint can be used as an alternative to exercise involving the affected joint, if or when exercising involving the injured joint is not practical or desired from a patient and clinical perspective. Current “rescue” exercise programmes for acute increases in knee pain have not always been effective (only 63% of patients reported decreased pain) (37); therefore, there is a clear need for alternative exercises that can be beneficial for knee pain while also avoiding knee movement. However, it should be noted that the inflammatory responses which may be responsible for acute pain experienced in the 24 h after exercise involving the joint are not necessarily detrimental to patients, from a physiological perspective. This inflammatory response may be necessary to mediate recovery and adaptation to exercise, as well as regulating aberrant neuroimmune process which are known to contribute to chronic pain (38, 39). Taking this into account, UB may only be effective as a short-term alternative for LB exercise, and it should not necessarily be advised that those with chronic knee pain who suffer from acute pain flares totally irradicate LB exercise from their exercise programmes and pain management, until more long-term and mechanistic research is conducted. However, the aerobic UB exercise used in this study may produce similar inflammatory responses to LB aerobic exercise, and therefore the inflammatory response may also be effective for mediating recovery and adaptations to exercise and regulating aberrant neuroimmune processes. Our data also suggest that the effects of a single bout of UB exercise may reduce VAS pain over several days as reflected in the moderate to large effect sizes presented in Table 3, although the study was not sufficiently powered to detect VAS over this timescale.

**Table 3:** Delta change VAS values from both exercise trials over 7 days.

		Pre	Post	Day 1	Day 2	Day 3	Day 4	Day 5	Day 6	Day 7
VAS (0–10)	UB	4.4 ± 1.7	−0.4 ± 0.8	−1.4 ± 1.7	−1.2 ± 1.6	−1.6 ± 1.6	−1.4 ± 1.7	−1.2 ± 1.5	−1.4 ± 1.7	−1.3 ± 2.0
	d	–	0.28	0.76	0.62	0.88	0.77	0.61	0.73	0.66
	LB	4.0 ± 1.9	−0.4 ± 2.1	+0.1 ± 2.1	−0.5 ± 1.9	−0.4 ± 1.5	−0.5 ± 1.8	−0.4 ± 1.7	−0.4 ± 1.6	−0.5 ± 1.7
	d	–	0.13	0.04	0.24	0.19	0.21	0.23	0.23	0.24

UB, upper body trial; LB, lower body trial.

*d* represents Cohen's d effect size for change in VAS pain from baseline.

### 4.2. Limitations

Although our results may have some clinical implications, it is important to highlight that this is the first study of its kind, and our results did not capture any mechanistic reasoning to support the findings. Further research is required to determine the potential mechanisms of the effect of UB aerobic exercise on knee pain. The minimal clinically important difference (MCID) for VAS pain in the knee OA population has been reported to be 19.9 mm on a 0–100 VAS scale (37) which translates to approximately 2 on a 0–10 VAS scale. VAS pain was only reduced by −1.4 ± 1.7 at 1 day post-exercise following UB exercise in this study; therefore, it did not quite meet the MCID for this population. Ten out of 19 (47%) participants in this study reported chronic knee pain in both knees; thus it is difficult to determine whether or not widespread hyperalgesia is present. It should also be noted that this population was not all clinically diagnosed with knee OA (eight out of 19 reported clinical knee OA diagnosis) and therefore it would be beneficial to use this protocol in a clinically diagnosed knee OA population. Furthermore, the age limit of 45–69 years for the inclusion criteria does not fully capture the breadth of the chronic knee pain population which may affect the generalisability of these results. The generalisability of results may also be affected by the fact that our sample size calculation was based upon a single study, potentially introducing selection bias. As this was the first study of its kind, we did not recruit a diverse-enough population to be able to split participants for analysis according to those who do positively respond to exercise involving the knee joint and those who are aggravated by exercise involving the joint, and future studies should look to incorporate this. Furthermore, outcome assessors were not blinded to allocation sequence which could have influenced PPT and MDT results. Future studies should also aim to determine the mechanisms by which UB exercise may be more effective in improving knee pain than LB exercise. In addition, although the knee pain-relieving benefits are apparent following just a single bout of UB exercise, we do not know how UB exercise training over a longer term would affect knee pain.

## 5. Conclusion

To conclude, a single bout of upper-body aerobic exercise was effective in reducing symptomatic pain at 24 h/1 day post-exercise compared with LB aerobic exercise in our participants with chronic knee pain. These results indicate that UB aerobic exercise may serve as an option for effective alternative therapeutic exercise for reducing knee pain in the short term, while avoiding acute increases in pain which are common for some patients following lower-body exercise in this population. More research is required to determine the mechanisms for this response and to examine the long-term effects of UB aerobic exercise on knee pain.

## Data Availability

The raw data supporting the conclusions of this article will be made available by the authors, without undue reservation.
